# A nomogram model for assessing predictors and prognosis of postoperative delirium in patients receiving acute type A aortic dissection surgery

**DOI:** 10.1186/s12872-023-03111-3

**Published:** 2023-02-07

**Authors:** Jian-Ling Lin, Guo-Zhong Zheng, Liang-Wan Chen, Zeng-Rong Luo

**Affiliations:** 1 Department of Cardiovascular Surgery and Cardiac Disease Center, Union Hospital, Fujian Medical University, Fuzhou, 350001 People’s Republic of China; 2 Key Laboratory of Cardio-Thoracic Surgery (Fujian Medical University), Fujian Province University, Fuzhou, People’s Republic of China

**Keywords:** Acute type A aortic dissection, Postoperative delirium, Risk factors, Outcomes, Nomogram

## Abstract

**Background:**

Postoperative delirium (POD) complicates the postoperative course. There is limited information on POD-related risk factors (RFs) and prognosis in patients with acute type A aortic dissection (ATAAD) after modified triple-branched stent graft implantation (MTBSG) surgery.

**Methods:**

We retrospectively examined consecutive ATAAD patients who received MTBSG surgery in our hospital between January 2013 and December 2019. We employed univariate and multivariate analyses to identify stand-alone RFs for POD. A nomogram was next generated to estimate POD occurrence. The primary outcome was the development of POD, and the secondary outcomes were intensive care unit (ICU) and hospital stays, hospitalization costs, and in-hospital and follow-up mortality.

**Results:**

We selected 692 patients, of whom 220 experienced POD (31.8%). Based on our analysis, the following factors enhanced the likelihood of POD development: alcohol consumption (*p* < 0.001), acute physiology and chronic health evaluation II score (*p* = 0.023), serum total bilirubin (*p* = 0.007), stage 3 acute kidney injury (*p* < 0.001), serum interleukin-6 (*p* = 0.031), post-operative analgesics usage (*p* = 0.015), and ventilation duration (*p* = 0.008). POD patients had significantly longer ventilator times (*p* = 0.003), ICU stays (*p* < 0.001), and hospital stays (*p* = 0.038), together with increased hospitalization costs (*p* < 0.001) and in-hospital mortality (*p* = 0.019). However, POD was not a RF for mortality during follow-up (log-rank *p* = 0.611).

**Conclusions:**

We demonstrated a strong link between POD and poor prognosis in ATAAD patients. We also constructed a prognosis estimator model which will benefit early management guidance to minimize the incidence of POD.

## Introduction

Acute type A aortic dissection (ATAAD) is a serious condition that often necessitates surgery [1]. Cerebral protection is of major concern during ATAAD of the aortic arch [2]. Even though deep hypothermic circulatory arrest (DHCA) with selective antegrade cerebral perfusion (SACP) is known to markedly reduce perisurgical damage to the nervous system, the incidence of postoperative delirium (POD) remains 12–37%, with grave patient outcomes [2, 3].

POD is a neuropsychiatric syndrome manifested by the acute development of dysregulated arousal and cognitive decline following anesthesia and operation [4]. The presence of POD significantly affects weaning from mechanical ventilation and is strongly associated with greater mobility, longer stay in the intensive care unit (ICU), increased hospital expenses, and worse patient prognosis [5].

A recent study reported a 32.5‒52.0% incidence of POD in AAD52.0% patients following Sun’s procedure [6]. These authors found the respective durations of surgery, cardiopulmonary bypass (CPB), and selective cerebral perfusion (SCP) to be risk factors (RFs) for POD following AAD. Unfortunately, even though modified triple-branched stent graft implantation (MTBSG) considerably reduces the duration of surgery, CPB, and aortic cross-clamping due to a reduction in vascular anastomosis of the aortic arch branches, neurological dysfunction remains among the most common and severe complications of MTBSG [7].

Previously, we observed a POD incidence of 37.86% following MTBSG [8]. Among the stand-alone RFs of POD were an acute physiology and chronic health evaluation II (APACHE-II) score > 20, hypoxemia, and other forms of analgesics and sedatives [8]. However, there is limited information on the long-term survival of AAD patients who developed POD after MTBSG.

At present, the POD incidence is relatively high, and without early diagnosis and treatment, it may progress to accidental tracheal intubation, aspiration, hypoxia, and even death [5]. Therefore, early POD prediction is of the utmost importance.

Here, we identified stand-alone POD indicators in AAD patients, and, subsequently, constructed an estimation model to predict POD occurrence. We next compared the long-term survival of patients with and without POD development after MTBSG for the treatment of AAD.

### Methods and patients

The study was approved by ethics committee of the Fujian Medical University Union Hospital. The need for written informed consent was waived due to the retrospective nature of the study.

### Study population

**Fig. 1:**
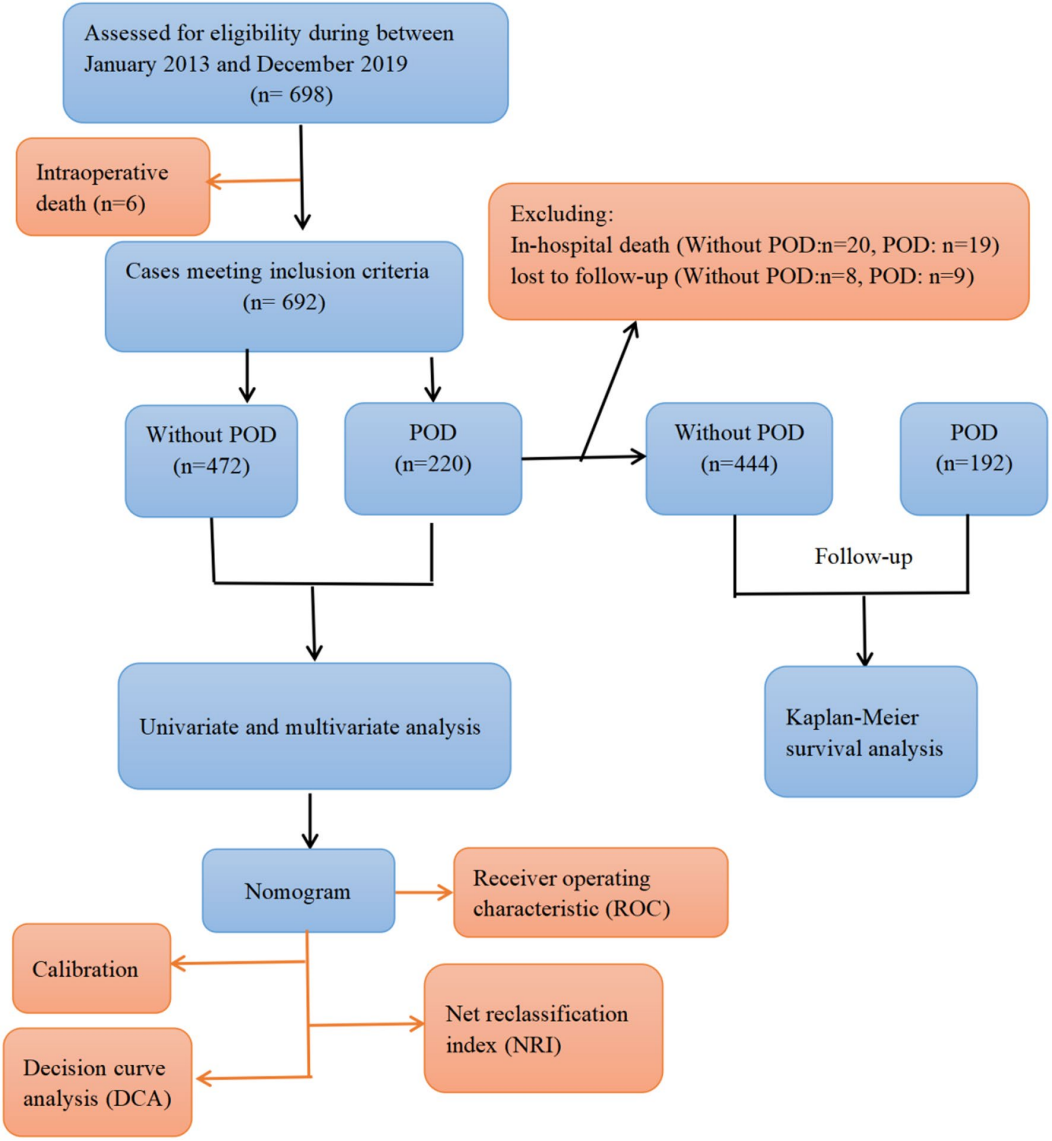
Flow chart of the study. (POD, postoperative delirium.)

Of the 698 patients who underwent MTBSG at our cardiac surgery center between January 2013 and December 2019, 692 survived the surgery and were consecutively enrolled in the study. The participants were assigned to two groups, namely, POD and non-POD sufferers (Fig. 1). The inclusion criteria were as follows: (1) age > 18 years, (2) documented permission to participate in the research. The exclusion criteria were as follows: (1) history of neurological or psychiatric disorders such as dementia, stroke, schizophrenia, and depression, (2) diagnosis of liver cirrhosis and uremia, (3) suffered from stroke or brain malperfusion prior to MTBSG, (4) suffered from cardiac tamponade-induced presurgical shock or hemodynamic instability, (5) levels of liver enzymes over four times those of baseline, (6) impaired hearing and/or vision, (7) patients who fell into coma after MTBSG, or who died < 24 h of MTBSG, (8) those who received extracorporeal membrane oxygenation therapy.

### Operative technique

All patients received a combination of intravenous and inhalational anesthesia. The anesthetic agents used were sevoflurane, midazolam, sufentanil, rocuronium, and dexmedetomidine, and dosages were based on patient body weight. Nasopharyngeal and rectal temperatures were recorded, as well as arm and leg blood pressures. The Regional Oximetry System (VISTA, Covidien) was used to measure local cerebral oxygen saturation. The depth of anesthesia was determined by the Bispectral Index (BIS) Monitoring System (VISTA, Covidien). The myocardium was protected through multiple perfusions of cold blood cardioplegia (4 °C) via the bilateral coronary arteries. The operation was conducted using a combination of low-flow CPB and cardiocirculatory arrest under deep hypothermia. The right axillary artery and the left carotid artery were utilized during bilateral SCP. The subsequent interventions were performed as described in our prior publication [7].

### Delirium evaluation

POD was assessed twice daily starting from postoperative day 4 while the patients remained in the ICU or the general ward. This was done in two steps: the Richmond agitation-sedation score (RASS) evaluated the degree of consciousness [9] with RASS values of < -4 discarded from the analysis. If the patient was sedated during the first RASS evaluation, the sedative doses were adjusted prior to a second evaluation 30 min later. The confusion assessment method for the intensive care unit (CAM-ICU) assessment was carried out when the RASS scores were > − 4 [10]. If there were alterations in the patient’s level of consciousness before sedative dosage adjustment, the CAM-ICU assessment was performed immediately.

The CAM-ICU identifies POD under the following circumstances: (1) acute onset and fluctuating course; (2) attention disorder; (3) disordered thinking; and (4) alternating consciousness. Delirium is typically diagnosed as either (1), (2), and (3) or (1), (2), and (4). All cognitive evaluations were conducted by two scientists who had training in CAM-ICU usage from a registered psychiatrist. All cognitive results were further verified by a third researcher with training in advanced cognitive examination. Lastly, all POD evaluators were blinded to the experimental information.

### Baseline data

The baseline information included patient information, comorbidities, presurgical laboratory data, and operation protocol. Specifically, patient information included age, sex, body mass index (BMI), smoking and drinking habits, education level, and occupation. Among them, “smoking history” was defined as continuous smoking of at least one-third pack per week for more than 1 year, and “drinking history” was defined as continuous alcohol consumption of at least 14 units per week for more than 1 year. Comorbidities included hypertension, diabetes mellitus, hyperlipidemia, chronic obstructive pulmonary disease, coronary heart disease, moderate or severe AR, renal dysfunction, malperfusion syndromes, atrial fibrillation, prior cardiac surgery, prior general surgery, New York Heart Association (NYHA) category, pericardial effusion, left and right ventricular diameters, right atrial and left atrial diameters, and left ventricular ejection fraction. The preoperative laboratory information included white and red blood cell counts, hemoglobin, platelet count, serum creatinine, serum total bilirubin, serum albumin, serum globulin, D-dimer, and plasma interleukin-6 (IL-6). The surgical procedure included isolated MTBSG surgery, MTBSG in combination with coronary artery bypass grafting, MTBSG in combination with other forms of cardiac surgery, MTBSG in combination with valve surgery, MTBSG in combination with valve and coronary surgery, cardiopulmonary bypass duration, DHCA duration, aortic cross clamp duration, lowest temperature, as well as red blood cell and platelet transfusion. Postsurgical information included the APACHE-II score, acute kidney injury (AKI), and analgesic and sedative use, as well as ventilation duration.

### Endpoints

The primary endpoint was in-hospital mortality evaluation on patients following MTBSG. The secondary endpoints included duration of ventilation, ICU or hospital stay, hospitalization costs, and death within median follow-up duration of 4.8 (2.6,6.7) years.

### Derivation and validation of the estimation model

We developed a risk estimation model using clinical information to estimate delirium risk in patients following MTBSG. First, we used univariate analysis to identify POD-related RFs in the patient population. The significant (*P* < 0.10) RFs from univariate analysis were then incorporated into the multivariate analysis to establish the stand-alone POD RFs. The results are provided as odds ratios (ORs) with 95% confidence intervals (CIs). Next, we generated a nomogram, using the stand-alone POD indicators and corresponding beta-coefficients, and performed an internal validation using the bootstrap (1000 replications) method. To assess the estimation precision, we performed calibration, discrimination, and clinical utility analyses of the nomogram. The area under the receiver operating characteristic (ROC) curve (AUC) was used for the assessment of prediction discrimination. The calibration curve determined agreement between the nomogram-based estimation and actual events. Decision curve analysis was used to evaluate the clinical utility of the nomogram through quantification of the standardized net benefits at varying risk thresholds.

### Statistical analysis

All data analyses were performed with SPSS (version 26.0) and R (version 4.1.3).

### Perioperative data analyses

The Shapiro–Wilk test was used to assess whether the data were normally distributed. Categorical and continuous variables were expressed as n (%) and mean ± standard deviation or median (interquartile range), respectively and were analyzed by descriptive analyses. Categorical data were analyzed using Chi-square or Fisher exact tests. Non-normally distributed variables were analyzed using the Mann–Whitney U test while whereas, normally distributed variables were analyzed with Student’s *t*-tests. Potential selection bias was minimized with propensity score matching (PSM) analysis using nearest available matching (1:1, 0.25). Lastly, a *p*-value < 0.05 was set as the significance threshold.

### Time-to-event analyses

The Kaplan–Meier curves were established for the death and neural complication within a median follow-up duration of 4.8 (2.6,6.7) years. Neural complication was defined as coma, disorientation, convulsions, hemiplegia, severe limb muscle dysfunction, etc. Multivariate analysis was used to identify stand-alone RFs for POD occurrence, as previously described.

## Results

### Baseline features

Overall, 698 patients received MTBSG surgeries during the 7-year investigation period. After the exclusion of 6 patients who expired during surgery, 692 patients were enrolled and analyzed. Of these, 220 patients (31.8%) experienced POD after MTBSG. Figure 1 illustrates a summary of our study design.

Patients in the POD group were older (*p* < 0.001), consumed more alcohol (*p* < 0.001), had increased incidence of renal dysfunction (*p* = 0.002) and AKI stage 3 (*p* = 0.037), and increased use of both analgesics and sedatives (*p* < 0.001), together with elevated serum creatinine (*p* = 0.005), postoperative total bilirubin (*p* < 0.001), and IL-6 (*p* < 0.001) levels, higher APACHE-II scores (*p* < 0.001), prolonged DHCA duration (*p* = 0.006), and included greater numbers of manual workers (*p* < 0.001) (Table 1).

**Table 1:** Univariate analysis of possible risk factors for POD after AADS

Characteristic	Without POD *n* = 472	With POD *n* = 220	*χ*^2^ /Z/t	*P*
*Demographics*				
Age, (years), mean ± SD	50.55 ± 10.38	57.98 ± 11.88	7.967	< 0.001
Male, *n* (%)	368 (78.0)	179 (81.4)	1.046	0.306
Body mass index, (kg/m^2^), mean ± SD	25.26 ± 3.65	25.53 ± 4.04	0.844	0.399
Smoking history, n (%)	189(40.0)	89 (40.5)	0.011	0.918
Drinking history, *n* (%)	155 (32.8)	125 (56.8)	35.817	< 0.001
Education level, *n* (%)			2.485	0.289
Primary and below	225 (47.7)	119 (54.1)		
Middle school	160 (33.9)	66 (30.0)		
High school and above	87 (18.4)	35 (15.9)		
Nature of occupation, *n* (%)			39.13	< 0.001
Manual worker	242 (51.3)	168 (76.4)		
Non-manual worker	230 (48.7)	52 (23.6)		
*Underlying conditions*				
Hypertension, *n* (%)	348 (73.7)	167 (75.9)	0.375	0.540
Diabetes mellitus, *n* (%)	45 (9.5)	22 (10.0)	0.037	0.847
Hyperlipidemia, *n* (%)	34 (7.2)	20 (9.1)	0.743	0.389
Chronic obstructive pulmonary disease, *n* (%)	26 (5.5)	16 (7.3)	0.819	0.365
Coronary heart disease, *n* (%)	40 (8.5)	25 (11.4)	1.472	0.225
Moderate or severe AR, *n* (%)	140 (29.7)	75 (34.1)	1.375	0.241
Renal dysfunction ^*^, *n* (%)	106 (22.5)	74 (33.6)	9.743	0.002
Malperfusion syndromes, *n* (%)			1.709	0.789
Cerebral	14 (21.2)	17 (28.3)		
Myocardial	11 (16.7)	12 (20.0)		
Renal	25 (37.9)	17 (28.3)		
Iliofemoral	8 (12.1)	7 (11.7)		
Gastrointestinal	8 (12.1)	7 (12.7)		
Atrial fbrillation, *n* (%)	12 (2.5)	8 (3.6)	0.640	0.424
Cardiac surgery history, *n* (%)	33 (7.0)	13 (5.9)	0.283	0.595
General surgery history, *n* (%)	65 (13.8)	41 (18.6)	2.738	0.098
NYHA class, *n* (%)			1.144	0.766
1	239(50.6)	114(51.8)		
2	169(35.8)	74(33.6)		
3	40(8.5)	17(7.7)		
4	24(5.1)	15(6.8)		
Pericardial effusion (moderate or large), *n* (%), median (Q1, Q3)	102 (21.6)	53 (24.1)	0.531	0.466
Diameter of the left atrium (cm), median (Q1, Q3)	3.6 (3.0, 4.0)	3.6 (3.2, 4.0)	0.188	0.912
Diameter of the left ventricle (cm), median (Q1, Q3)	4.9 (4.4, 5.4)	4.8 (4.3, 5.4)	0.952	0.302
Diameter of the right atrium (cm), median (Q1, Q3)	3.8 (3.6, 4.0)	3.7 (3.6, 4.1)	0.273	0.785
Diameter of the right ventricle (cm), median (Q1, Q3)	3.8 (3.5, 4.1)	3.7 (3.5, 3.9)	0.877	0.386
Left ventricular ejection fraction (%), median (Q1, Q3)	60 (53; 65)	59 (52; 64)	0.440	0.575
*Preoperative Laboratory values*				
White blood cell count (× 10^9^/L), median (Q1, Q3)	8.8 (6.7, 12.0)	9.1 (7.0, 13.7)	1.748	0.080
Red blood cell count (× 10^12^/L), median (Q1, Q3)	5.0 (3.9, 5.6)	4.9 (4.0, 5.3)	1.006	0.240
Hemoglobin (g/L), median (Q1, Q3)	122 (110, 140)	119 (115, 140)	0.232	0.807
Platelet count (× 10^9^/L), median (Q1, Q3)	172 (133, 220)	158 (129, 199)	1.386	0.112
Serum creatinine (μmol/L), median (Q1, Q3)	89.2 (77.0, 118.6)	106.5 (89.9, 128.8)	2.840	0.005
Serum total bilirubin (umol/L), median (Q1, Q3)	28.7 (23.6, 36.6)	29.6 (26.9, 37.0)	0.364	0.701
Serum albumin (g/L), median (Q1, Q3)	38.5 (35.8, 42.5)	38.0 (34.5, 41.0)	0.288	0.796
Serum globulin (g/L), median (Q1, Q3)	24.0 (22.0, 27.9)	24.5 (22.5, 28.5)	0.479	0.656
D-Dimmer (ug/L), median (Q1, Q3)	182(48,372)	198(38,309)	0.552	0.588
IL-6 (pg/ml), median (Q1, Q3)	53.15 (31.85, 95.60)	118.42 (59.85, 169.60)	9.866	< 0.001
*Intraoperative data*				
Catogeries of surgery, n (%)			1.291	0.863
Isolated AAD surgery	309 (65.5)	143 (65.0)		
Combined valve surgery	105 (22.2)	44 (20.0)		
Combined coronary artery bypass grafting	26 (5.5)	14 (6.4)		
Combined valve and coronary surgery	26 (5.5)	15 (6.8)		
Combined other types of cardiac surgery	6 (1.3)	4 (1.8)		
Bispectral Index (BIS)	40.54 ± 5.75	39.98 ± 5.88	1.184	0.237
Deep hypothermic circulatory arrest (mins), median (Q1, Q3)	14.5 (12.0, 20.0)	18.5 (15.0, 24.5)	2.766	0.006
Cardiopulmonary bypass time (minutes), median (Q1, Q3)	148 (104, 176)	149 (105, 178)	0.308	0.768
Aortic cross clamp time (minutes), median (Q1, Q3)	59.5 (48.5, 69.2)	61.8 (51.9, 70.7)	1.039	0.207
Lowest temperature (°C), mean ± SD	23.20 ± 2.85	23.29 ± 2.58	0.406	0.688
Transfusion of red blood cells (units), mean ± SD	4.50 ± 3.85	4.84 ± 3.90	1.077	0.282
Transfusion of platelet (units), mean ± SD	8.88 ± 3.60	9.80 ± 3.70	3.103	0.002
*Postoperative data*				
APACHE-II, mean ± SD	10.80 (3.50)	18.89 (3.95)	25.60	< 0.001
Serum total bilirubin (umol/L), median (Q1, Q3)	53.83(35.59, 87.57)	106.68(65.50, 138.58)	8.985	< 0.001
AKI, *n* (%)			8.481	0.037
Stage 1	36 (7.6)^aa^	9(4.1)^aa^		
Stage 2	23(4.9)^aa^	12(5.5)^aa^		
Stage 3^#^	57(12.1)^ab^	42(19.1)^ab^		
Analgesics and sedatives, n (%)	202 (42.8)	158 (71.8)	50.64	< 0.001
Ventilation time, h, median (Q1, Q3)	40.0 (28.4, 63.6)	60.0 (39.0, 115.7)	3.203	0.003
ICU stay time, h, median (Q1, Q3)	108.9(82.5, 170.5)	166.5(116.5, 206.2)	6.858	< 0.001
Hospital time, d, median (Q1, Q3)	17 (15, 22)	21 (15, 25)	2.763	0.038
Hospital costs, thousand dollars, median (Q1, Q3)	33.83 (30.55, 43.24)	39.80(33.65, 46.84)	6.018	< 0.001
In-hospital mortality, *n* (%)	20(4.2%)	19(8.6%)	5.460	0.019

* Defined as preoperative creatinine greater than 1.5 mg/dL

“Smoking history” was defined as continuous smoking of at least one-third pack per week for more than 1 year

“Drinking history” was defined as continuous alcohol consumption of at least 14 units per week for more than 1 year

Stage 3^#^ means the proportion of AKI stage 3 was significantly different between the two groups by Bonferroni method

“aa” represents no significant difference between the two groups; “ab” represents significant difference between the two groups

*Q1* First quartile, *Q3* Third quartile, *IL-6* Interleukin-6, *NYHA* New York Heart Association, *AKI* Acute kidney injury, *APACHE-II* Acute physiology and chronic health evaluation II, *ICU* Intensive care unit

### Construction and validation of the risk estimation model

**Fig. 2:**
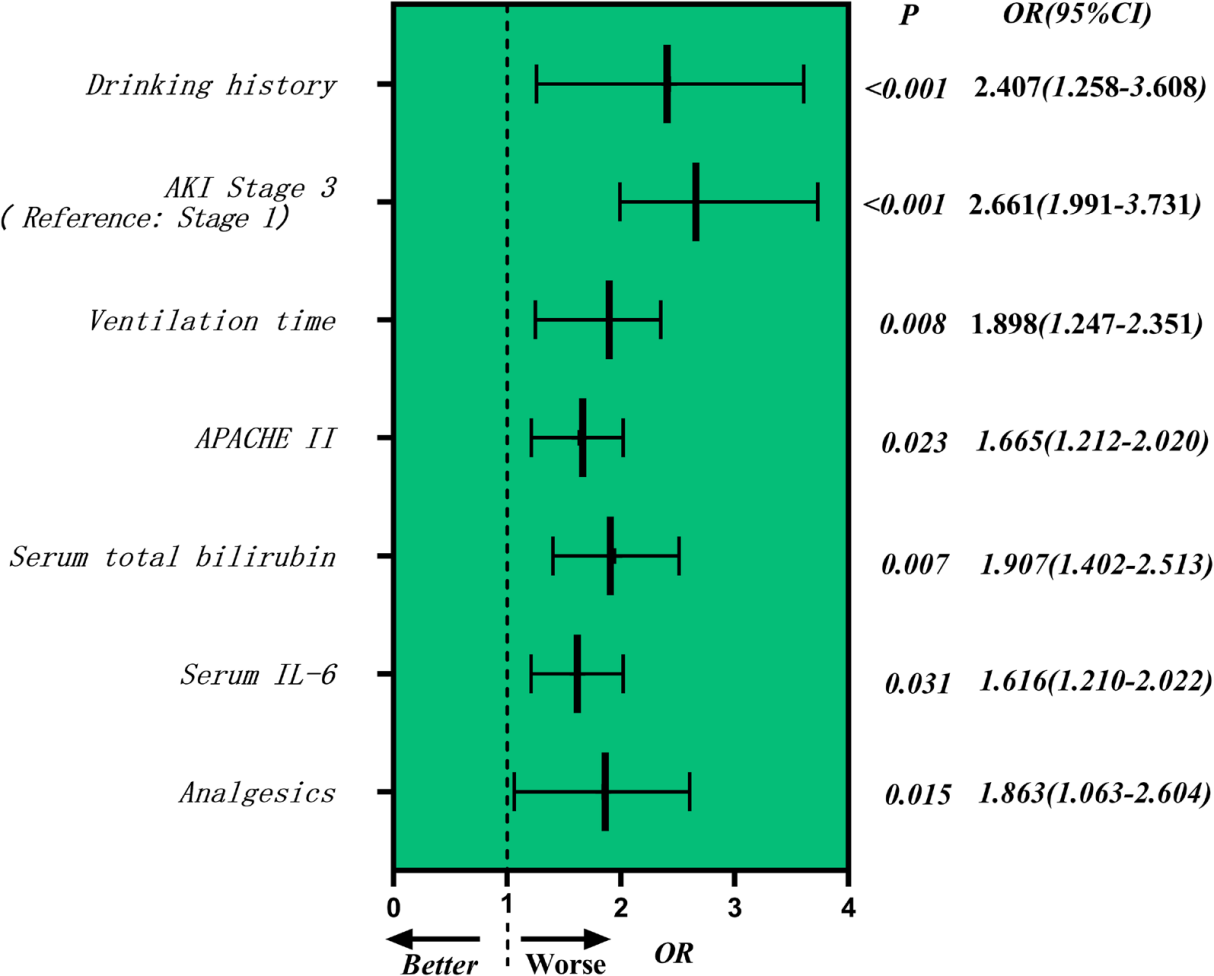
Multivariate logistic regression analysis identifed seven independent risk factors of the occurrence of POD in the forest plot, including alcohol consumption (OR 2.407, 95% CI 1.258–3.608, *p* < 0.001), APACHE II score (OR 1.665, 95% CI 1.212–2.020, *p* = 0.023), serum total bilirubin (OR 1.907, 95% CI 1.402–2.513, *p* = 0.007), AKI Stage 3 (Reference: Stage 1) (OR 2.661, 95% CI 1.991–3.731, *p* < 0.001), serum IL-6 (OR 1.616, 95% CI 1.210–2.022, *p* = 0.031), post-operative analgesics use (OR 1.863, 95% CI 1.063–2.604, *p* = 0.015), and ventilator time (OR 1.898, 95% CI 1.247–2.351, p = 0.008). (*POD* Postoperative delirium, *AKI* Acute kidney injury, *IL-6* Interleukin-6, *APACHE-II* Acute physiology and chronic health evaluation II, *CI* Confidence interval, *OR* Odds ratio.)

Univariate analysis was used to identify 15 putative RFs for POD development in patients who received MTBSG surgery (Table 1). We then conducted a collinearity diagnostic assessment before the construction of the multivariate model. Using stepwise forward selection, we identified 7 stand-alone RFs that accurately estimated POD occurrence. These included alcohol consumption (OR 2.407, 95% CI 1.258–3.608, *p* < 0.001), APACHE II score (OR 1.665, 95% CI 1.212–2.020, *p* = 0.023), postoperative serum total bilirubin (OR 1.907, 95% CI 1.402–2.513, *p* = 0.007), AKI Stage 3(Reference: Stage 1) (OR 2.661, 95% CI 1.991–3.731, *p* < 0.001), serum IL-6 (OR 1.616, 95% CI 1.210–2.022, *p* = 0.031), post-operative analgesic usage (OR 1.863, 95% CI 1.063-2.604, *p* = 0.015), and ventilation duration (OR 1.898, 95% CI 1.247-2.351, *p* = 0.008) (Figure. 2).

**Fig. 3:**
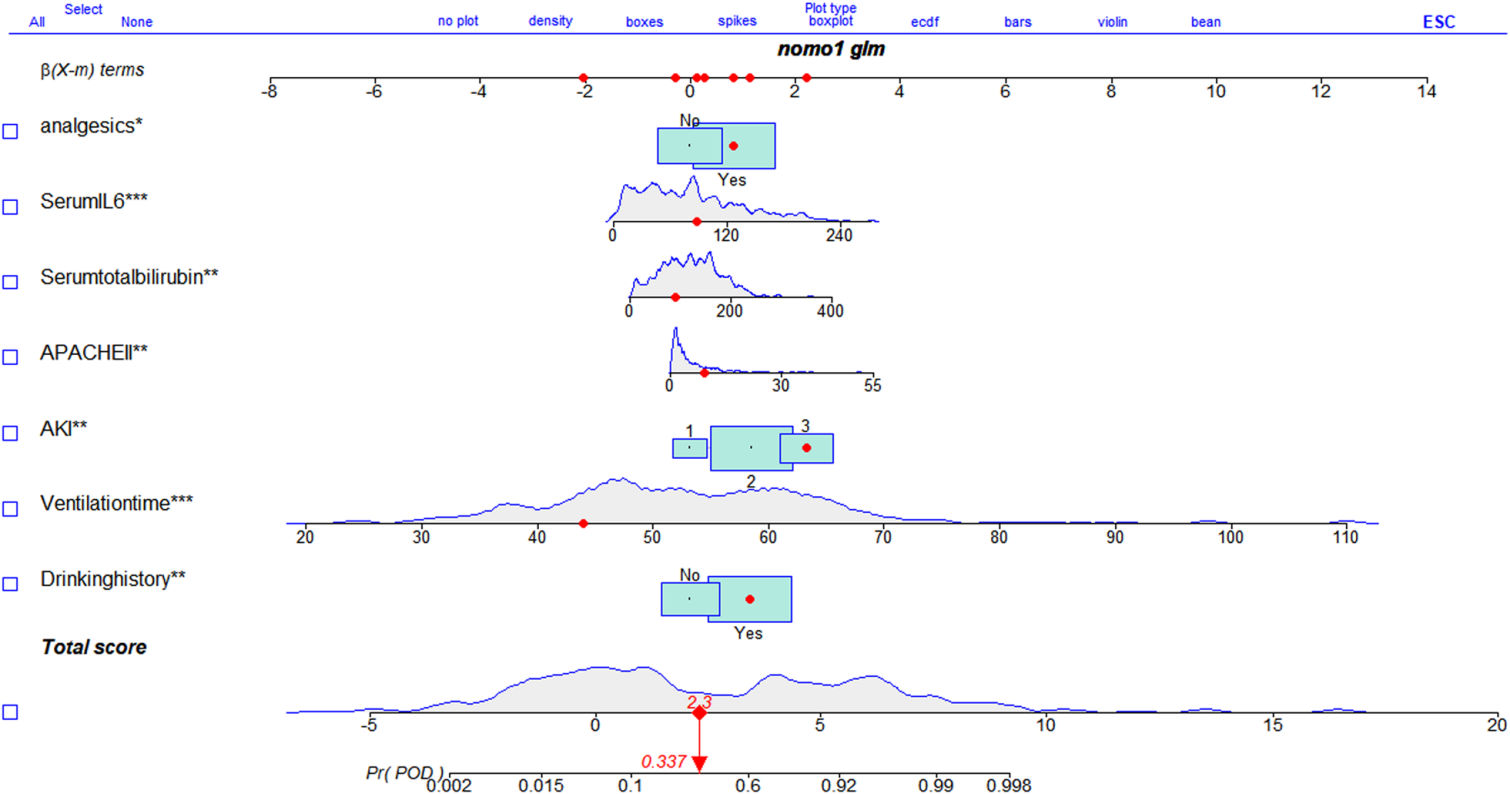
Nomogram for predicting POD. Each red dot represents each variable beta-coefcients of the patient. The total point is 2.3, corresponding to a probability of 33.7% to develop POD. (POD, postoperative delirium.)

A nomogram based on the results of the multivariate analysis was then constructed. The regression coefficient of each predictor was scaled between 0–100 points, reflecting the relative significance of each predictor (Figure. 3). The POD risk was then computed by the addition of the scores of all the variables. The POD scores in the nomogram ranged from 0.002 to 0.998.

**Fig. 4:**
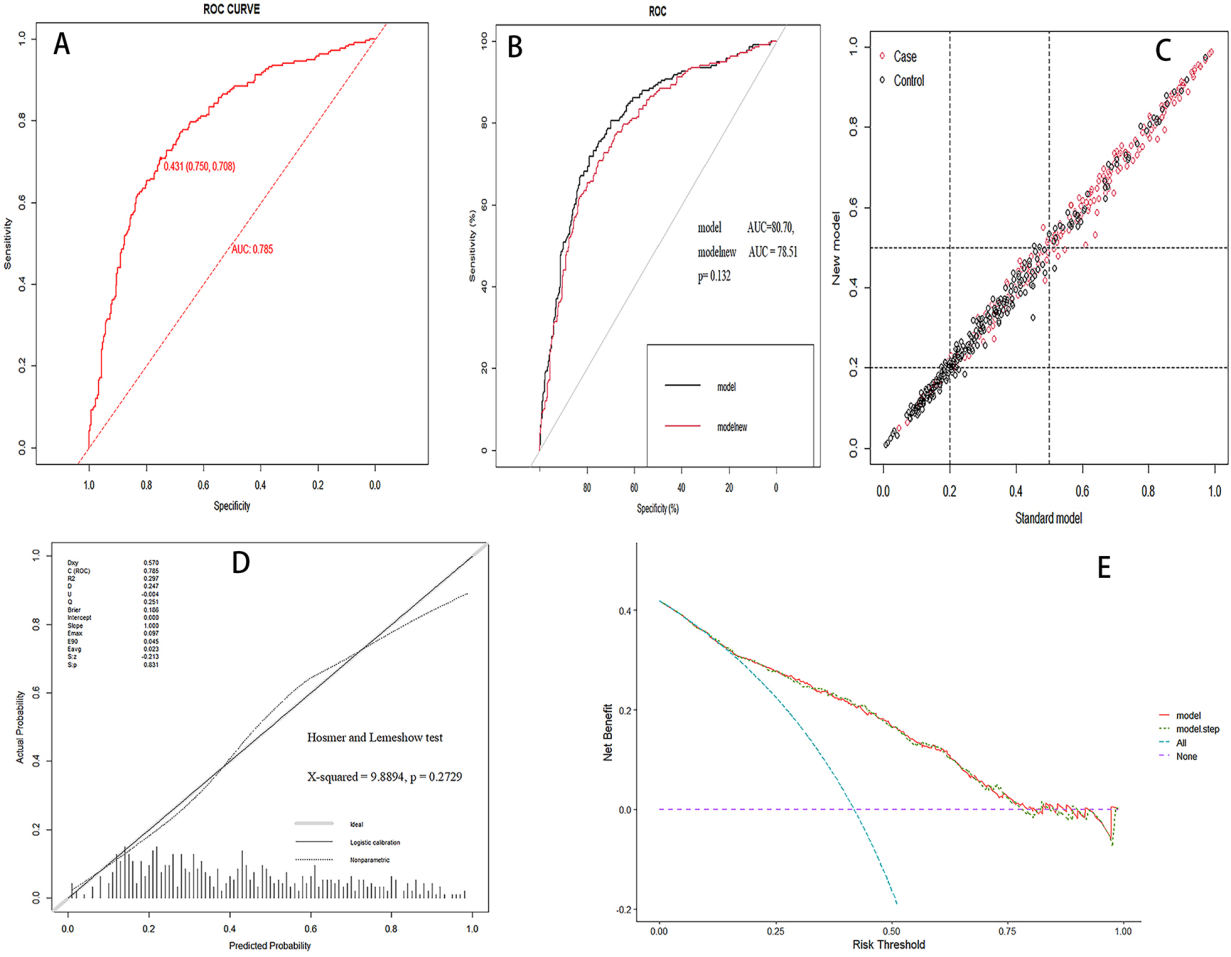
Assessment and validation of the nomogram. **A.** ROC curve for the nomogram; **B.** DeLong's test was used to evaluate the two correlated ROC curves of model (*p* = 0.132); **C.** Calculation of the NRI using a cut of 0.05 showing good efficacy; **D.** Calibration plot of the nomogram. Ideal line represents perfect prediction that nomogram-predicted probability matches actually observed probability; and **E.** The decision curves showed the nomogram model was clinically useful. (*ROC* Receiver operating characteristic, *AUC* Area under the receiver operating characteristic curve, *NRI* Net reclassification index.)

We next internally validated our model using a bootstrap technique containing 1,000 resamples from our patient population. The AUC was 0.785 (Fig. 4A). We then used the DeLong test to assess the two associated ROC curves of the model (*p* = 0.132) (Fig. 4B), which indicated good discrimination of the nomogram. Calculation of the net reclassification index (NRI) using a cut of 0.05 showed good efficacy (Fig. 4C). Calibration with the Hosmer and Lemeshow goodness-of-fit (GOF) test was used for model evaluation (*χ*^2^=9.8894, *p*=0.2729). Visual observation showed good calibration (Fig. 4D). Based on the decision curve analysis of the “intervention for all” and “no intervention” approaches, the estimation model demonstrated additional clinical benefit when the risk threshold was adjusted to 0.2-0.50 (Fig. 4E).

## Secondly endpoint

### Outcomes

Of the 692 patients, 220 patients who experienced POD also had prolonged ventilator time (60.0 (39.0, 115.7) vs. 40.0 (28.4, 63.6) hours, *p* = 0.003), ICU stay (166.5 (116.5, 206.2) vs. 108.9 (82.5, 170.5) hours, *p* < 0.001) and hospital stay (21 (15, 25) vs. 17 (15, 22), *p* = 0.038), and increased hospitalization costs (39.80 (33.65, 46.84) vs. 33.83 (30.55, 43.24) thousand dollars, *p* < 0.001), relative to non-POD patients. The POD patients also showed significantly elevated in-hospital mortality rates (8.6% (*n* = 19) vs. 4.2% (*n* = 20), *χ*^2^ = 5.460, *p* = 0.019) (Table 1). Next, we conducted propensity score matching analysis, and retrieved 135 patients from each group (Table 2). Other than hospitalization costs, POD was still significantly associated with prolonged ventilation time (59.2 (39.3,113.0) vs. 42.5 (29.0, 64.2) hours, *p* = 0.016), ICU stay time (160.0 (115.5,205.6) vs. 109.2 (86.5, 172.4) hours, *p* = 0.002), hospital stay time (21 (15, 24) vs. 17 (15, 23), *p* = 0.043), and in-hospital mortality rate (10.4% (*n* = 14) vs. 3.7% (*n* = 5), *χ*^2^ = 4.586, *p* = 0.032).

**Table 2:** Outcomes in patients with and without POD in patients undergoing AAD surgery after propensity score matching

Variables	All patients *n* = 270	Without POD *n* = 135	With POD *n* = 135	χ^2^ /Z	*P*
Ventilation time, h, median (Q1, Q3)	49.5 (35.3, 91.8)	42.5 (29.0, 64.2)	59.2 (39.3,113.0)	3.083	0.016
ICU stay time, h, median (Q1, Q3)	117.2 (92.5, 182.6)	109.2 (86.5, 172.4)	160.0 (115.5,205.6)	5.995	0.002
Hospital time, d, median (Q1, Q3)	18 (15, 24)	17 (15, 23)	21 (15, 24)	2.985	0.043
Hospital costs, thousand dollars, median (Q1, Q3)	35.82 (32.30,40.02)	33.90 (31.25, 40.56)	37.80 (33.85,43.56)	1.386	0.098
In-hospital mortality, *n* (%)	19 (7.0%)	5 (3.7%)	14 (10.4%)	4.586	0.032

*Q1* First quartile, *Q3* Third quartile, *ICU* Intensive care unit

### Time-to-event

**Fig. 5:**
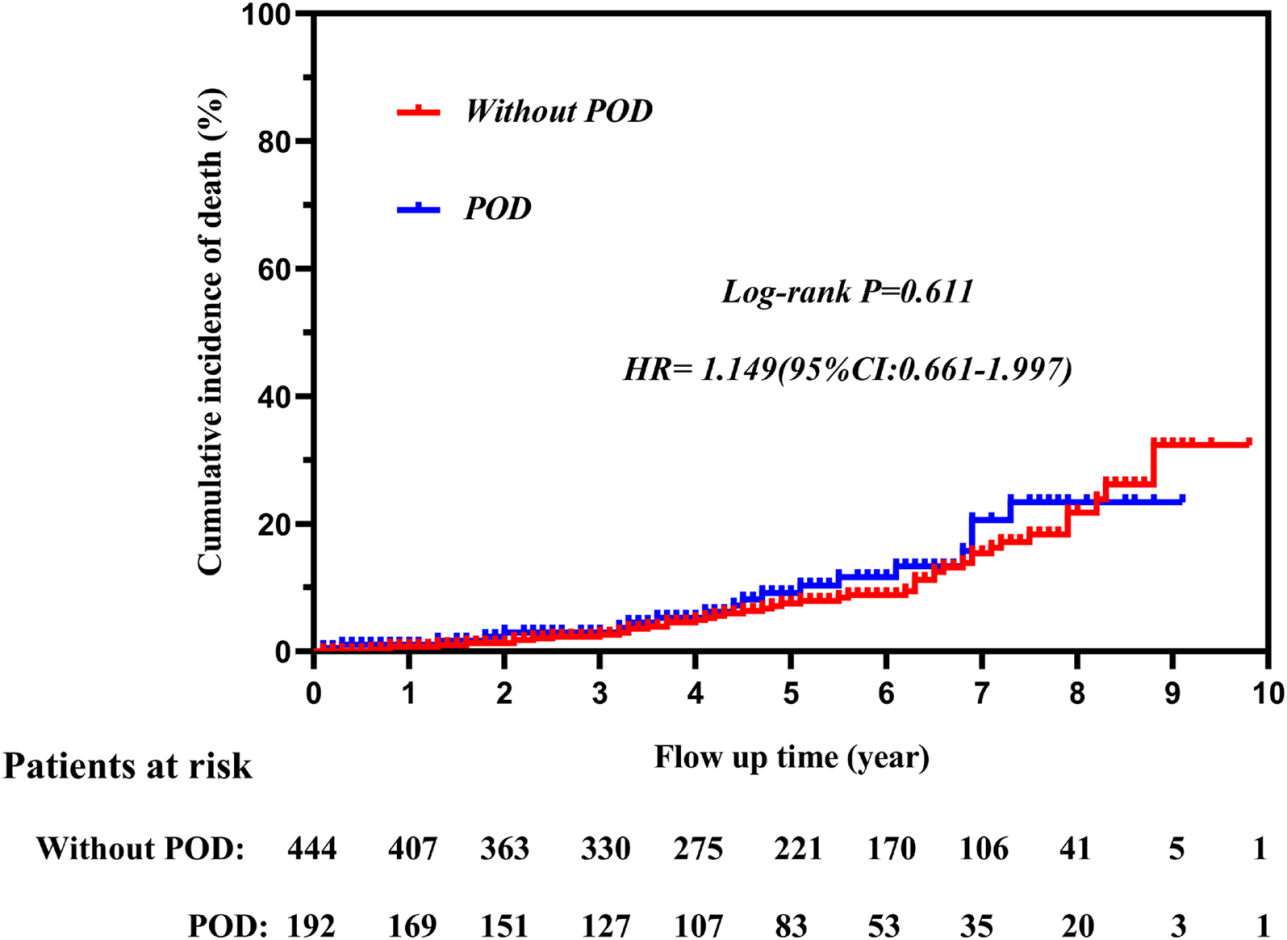
Survival curve of the follow-up patients withour POD and with POD. (POD, postoperative delirium.)

Six hundred and fifty-three patients (452 non-POD and 201 POD patients) remained after the exclusion of in-hospital deaths. Of these, 17 patients were lost to follow-up (8 non-POD and 9 POD patients). Hence, 636 patients completed the follow-up (444 non-POD and 192 POD patients). Interestingly, unlike in-hospital mortality, POD was not associated with long-term cumulative mortality and neural complications. Briefly, the cumulative incidence of mortality was 10.1% (*n* = 45) for POD and 9.9% (*n* = 19) for non-POD patients, with an HR of 1.149, (95%CI 0.661–1.997, log-rank *P* = 0.611) (Fig. 5); in addition, the incidence of neural complication was 3.13% (*n* = 6) for POD and 2.70% (*n* = 12) for non-POD patients, with an HR of 0.708, (95%CI 0.247–2.025, log-rank *P* = 0.486).

## Discussion

In this study, we observed a POD rate of 31.8% among AAD patients who underwent MTBSG implantation, which corroborates prior conclusions [6, 8]. Moreover, we demonstrated that POD patients experienced prolonged ventilation durations, ICU stays, and in-hospital mortality after MTBSG implantation, relative to non-POD patients. Based on our observations, the in-hospital mortality among AAD patients with POD following MTBSG implantation was 8.6%, which is similar to the reported incidence [6, 11]. This emphasizes the necessity for the early identification of high-risk patients who may develop POD following MTBSG surgery. This is a novel study that examined the effect of POD on AAD patient prognosis, and we generated a nomogram model to estimate POD occurrence. In the 692 patients who received MTBSG at our hospital, the stand-alone RFs of POD development after MTBSG included alcohol consumption, APACHE II score, serum total bilirubin, AKI Stage 3(Reference: Stage 1) , serum IL-6, post-operative analgesic usage, and ventilation duration. The estimation model was based on these factors and was found to exhibit good estimation performance and clinical utility.

Many studies have reported alcohol consumption to be a stand-alone RF for POD [12]. Regular alcohol consumption induces the release of neurotransmitters, such as dopamine, pentahydroxy tryptophan, opioids, and norepinephrine. These activate the mechanisms of reinforcement and reward in the brain which, in turn, generate a dependence on alcohol [13]. Brain receptors bind alcohol to inhibit excitatory nerve pathway activity, while also interfering with the communication between nerve cells. Neurodegeneration, neurocognitive deficits, and neuronal injury are well documented in alcoholics. [14] Thus, chronic excessive drinking can adversely affect both memory and cognition [14]. Herein, we presurgical alcohol consumption on POD development in AAD patients who receive MTBSG implantation. We discovered that alcohol consumption was markedly elevated among the POD patients relative to non-POD patients (P<0.001). Multivariate analysis showed that alcohol consumption was a stand-alone RF for POD development (OR 2.407, 95% CI 1.258–3.608, *p* < 0.001) and perioperative alcohol consumption could have played a role in inducing POD. Therefore, preoperative instructions for alcoholic patients to stop drinking, and to abstain from alcohol intake may be critical for the prevention of POD.

Previous investigations have reported that the APACHE II score is a stand-alone indicator of POD [15]. This study used the acute physiology age and chronic health evaluation scale, APACHE II, to evaluate the patient's condition. This scale assesses both the severity and prognosis of disease [16]. In our study, POD also provided predictive value of in-hospital mortality, which corroborated a prospective investigation in The Netherlands that examined the relationship between POD and mortality estimation, using the APACHE II scale [17].

We also found that the duration of ventilation also strongly predicted POD risk in our patient population. Based on a recent study based meta-analysis, prolonged mechanical ventilation (per hour) is significantly correlated with enhanced POD risk [18]. Even though the endotracheal tube is not inherently deliriogenic, extubation often follows a reduction in sedative drug administration, diminished risk of infection (ventilator-acquired pneumonias), enhanced mobility, and augmented environmental association, which may synergistically limit the development of POD [19]. Moreover, during mechanical ventilation, it is crucial to sustain a state of analgesia or conscious sedation, which requires the use of medications. Unfortunately, these medications sometimes impair neuronal function, thereby resulting in POD [6]. Hence, early discontinuation of sedative drugs may contribute to relatively low POD incidence. [20]

**Fig. 6:**
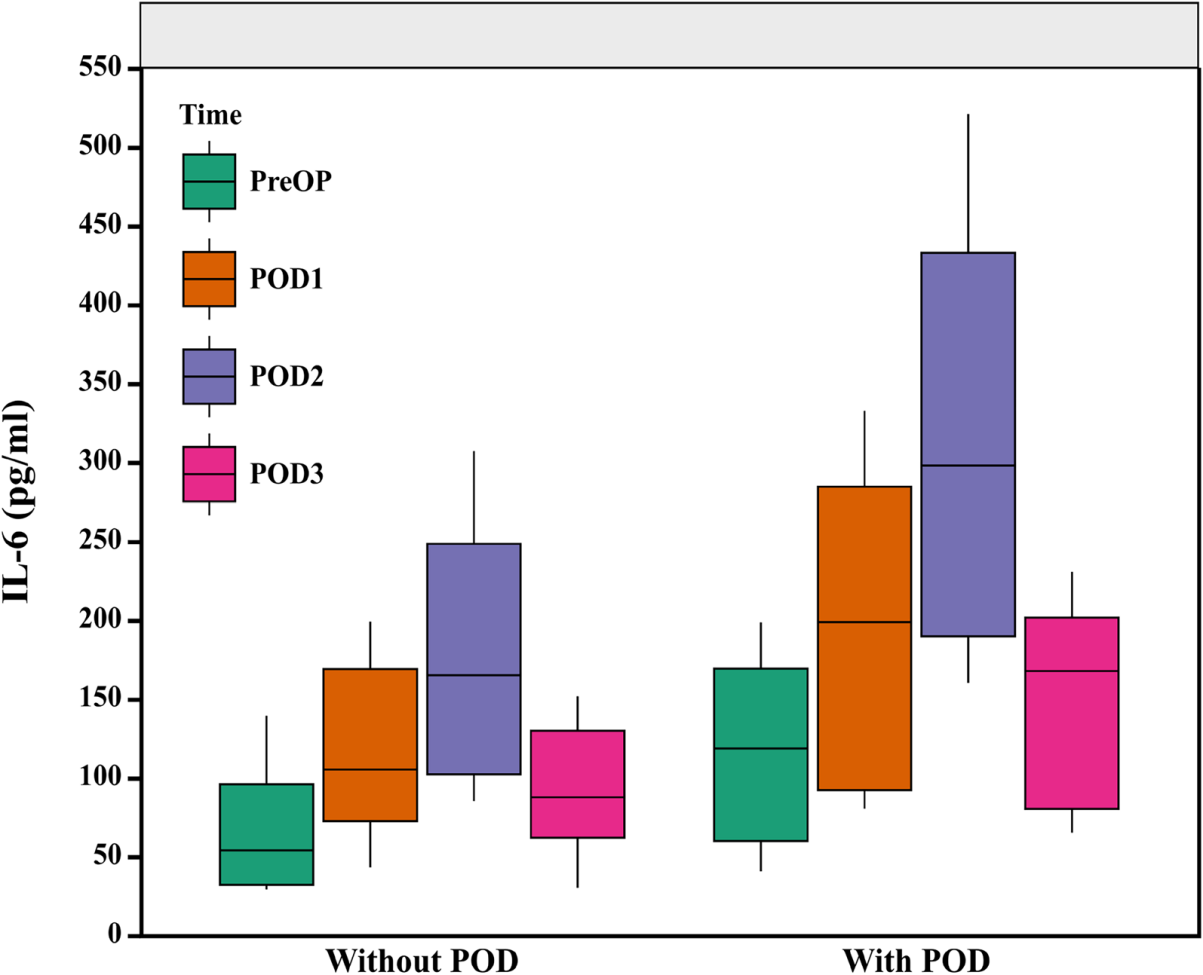
Daily comparison of the IL-6 levels between the without-POD and with-POD cohorts. (with POD, patients with postoperative delirium; without *POD* Patients without postoperative delirium, *PreOP* Preoperative, *POD1* Postoperative day 1, *POD2* Postoperative day 2, *POD3* Postoperative day 3, *IL-6* Interleukin-6.)

IL-6 is a proinflammatory agent released from damaged or inflamed tissues, and it regulates local and systemic processes to elicit multiple physiological functions [21]. Prior investigations have shown that early postoperative increases in chemokines are strongly association with the development of POD after cardiac surgery [22]. It was speculated that systemic inflammation impairs blood brain barrier (BBB) integrity, which, in turn, induces endothelial dysfunction and translocation of peripheral cells and corresponding factors into the brain parenchyma, thereby increasing the POD risk [23]. Here, we observed elevated inflammatory factor concentrations in POD patients compared with non-POD patients. This verified that the plasma IL-6 values can function as predictors of POD occurrence in AAD patients following MTBSG implantation [24]. Notably, in our study, the postoperative plasma IL-6 levels peaked on the second postoperative day (Fig. 6), which was consistent with an earlier report that showed increased incidence of delirium on the second postoperative day [8].

Hyperbilirubinemia is associated with preoperative hyperbilirubinemia, hemolysis, cardiotomy suction, gaseous micro-emboli, release of inflammatory factors, and blood transfusions during CPB [25]. A multivariate analysis by Dubois et al. found that elevated total serum bilirubin was an independent stand-alone RF for POD [26]. Bilirubin is the final lipid-soluble product of heme metabolism in mammals and readily traverses cell membranes such as the BBB, and is known to possess both neuroprotective and neurotoxic properties [27, 28]. Bilirubin crosses the BBB and associates with nerve cells to exert cytotoxic functions. The main bilirubin-based neurotoxic networks involve cell membrane perturbation, DNA damage, synaptic transmission modification, enhanced cytokine secretion, neurotransmitter axis suppression, and apoptosis [27, 29]. A typical feature of POD is the time delay between surgery and delirium onset. Thus, early detection and prevention of POD are crucial to the treatment of AAD patients who are at risk of POD development. This form of management can potentially reduce the morbidity and mortality of these patients.

The predictors that were found to be significantly related to the development of POD in our patient cohort included acute kidney injury (AKI). Previous studies [30] have indicated that higher AKI stages are a potential risk factor for POD development. Multiple pathophysiological mechanisms explain the enhanced delirium risk in patients with advanced AKI stages. Many uremic toxins produce uremic encephalopathy and disturbances within BBB modulate acute and chronic uremic encephalopathy [31]. Moreover, AKI promotes inflammation and alterations in the functioning of the brain [31].

Another RF associated with POD was the use of sedative and analgesic drugs, which are commonly employed in ICUs, and is consistent with earlier publications that confirmed a direct link between POD and sedative and analgesic drug usage [32]. Sedative and analgesic medications primarily affect the central nervous system, including nerve cell membranes, neurotransmitters, and cerebral metabolism [33]. The primary role of the central muscarine cholinergic system is to preserve cognitive abilities, and a myriad of sedative and analgesic medications target the central alkaloid receptor [34]. Persistent sedative usage impairs the circadian rhythm and produces steady superficial sleep while reducing the amount of deep and rapid eye movement sleep and adversely affecting the quality of the sleep–wake rhythm in critically ill patients. [35] Not surprisingly, increased rates of POD have been reported in patients receiving morphine. Dubois et al. [26] demonstrated that a daily dose of 7.2–18.6 mg of morphine led to a nine-fold increase in the POD risk relative to those who were not administered morphine. Taken together, sedative and analgesic medication usage must be critically considered and monitored, according to the patient’s clinical condition.

Here, we demonstrated a strong correlation between POD and poor patient prognosis. This correlation was still present after restricting the influences of selection bias, preoperative variable heterogeneity, and surgical duration. Our conclusions on the patient clinical profile, prognosis, and RFs involving in-hospital mortality can potentially benefit clinicians in managing AAD patients after surgery. In the clinic, attention should be paid to the prevention of delirium, and we recommend a warning post hung on the wall to avoid accidental extubation. Additionally, clinicians must reinforce tracheal intubation, straighten all drainage channels, and conduct other preventive monitoring measures to avoid perioperative damage.

### Limitations

The study has some limitations. Firstly, the study involved a single institution and had a relatively small sample size. Hence, our estimation model may not be applicable in the general population. Moreover, due to the retrospective design and lack of external verification, our conclusions must be interpreted with caution, prior to verification with prospective studies.

## Conclusion

The POD incidence among AAD patients receiving MTBSG was found to be 31.8%. The incidence of POD was strongly associated with prolonged ICU and hospital stays as well as increased hospitalization costs and mortality. We identified 7 significant RFs for POD, and constructed an estimation model that accurately predicted the occurrence of POD in patients who had received surgery. This model can guide the early detection of POD and estimate the prognosis of AAD patients.

## Data Availability

The data that support the findings of this study are available from Fujian Cardiac Medical Center but restrictions apply to the availability of these data, which were used under license for the current study, and so are not publicly available. Data are however available from Zeng-Rong Luo author upon reasonable request and with permission of Fujian Cardiac Medical Center.

## Abbreviations

ATAAD: Acute type A aortic dissection
POD: Postoperative delirium
ROC: Receiver operating characteristic
SACP: Selective antegrade cerebral perfusion
ICU: Intensive care unit
IL-6: Interleukin-6
NYHA: New York heart association
APACHE-II: Acute physiology and chronic health evaluation II
AKI: Acute kidney injury
RFs: Risk factors
MTBSG: Modified triple-branched stent-graft
PSM: Propensity score matching
CI: Confidence interval
OR: Odds ratio
HR: Hazard ratio

## References

[1] Ji Q, Lai H, Sun YongXin, Luo Z, Liu L, Liu C (2017). Impact of presurgical mild acute respiratory distress syndrome on surgical mortality after surgical repair of acute type A aortic dissection. Int Heart J.

[2] Zierer A, Moon MR, Melby SJ, Moazami N, Lawton JS, Kouchoukos NT, Pasque MK, Damiano RJ (2007). Impact of perfusion strategy on neurologic recovery in acute type A aortic dissection. Annals Thor Surg.

[3] Kazui T, Yamashita K, Washiyama N, Terada H, Bashar AHM, Suzuki T (2002). Usefulness of antegrade selective cerebral perfusion during aortic arch operations. Ann Thorac Surg.

[4] Inouye SK, Westendorp RGJ, Saczynski JS (2014). Delirium in elderly people. Lancet.

[5] Mangusan RF, Hooper V, Denslow SA, Travis L (2015). Outcomes associated with postoperative delirium after cardiac surgery. Am J Crit Care.

[6] Shi Q, Mu X, Zhang C, Wang S, Hong L, Chen X (2019). Risk factors for postoperative delirium in type a aortic dissection patients: a retrospective study. Med Sci Monit.

[7] Chen L-W, Dai X-F, Xi-Jie Wu, Liao D-S, Yun-Nan Hu, Zhang H (2017). Ascending aorta and Hemiarch replacement combined with modified triple-branched stent graft implantation for repair of acute DeBakey type I aortic dissection. Ann Thorac Surg.

[8] Lin Y, Chen Q, Zhang H, Chen L-W, Peng Y, Huang X (2020). Risk factors for postoperative delirium in patients with triple-branched stent graft implantation. J Cardiothorac Surg.

[9] Sessler CN, Gosnell MS, Grap MJ, Brophy GM, O'Neal PV, Keane KA (2002). The Richmond agitation-sedation scale: validity and reliability in adult intensive care unit patients. Am J Respir Crit Care Med.

[10] Smulter N, Lingehall HC, Gustafson Y, Olofsson B, Engstrom KG (2015). Validation of the confusion assessment method in detecting postoperative delirium in cardiac surgery patients. Am J Crit Care.

[11] Cai S, Zhang X, Pan W, Latour JM, Zheng J, Zhong J, Gao J, Lv M, Luo Z, Wang C, Zhang Y (2020). Prevalence, predictors, and early outcomes of post-operative delirium in patients with type A aortic dissection during intensive care unit stay. Front Med.

[12] Sousa G, Pinho C, Santos A, Abelha FJ (2017). Postoperative delirium in patients with history of alcohol abuse. Rev Esp Anestesiol Reanim.

[13] Michalak A, Biała G (2016). Alcohol dependence–neurobiology and treatment. Acta Pol Pharm.

[14] Mukherjee S (2013). Alcoholism and its effects on the central nervous system. Curr Neurovasc Res.

[15] Gravante F, Giannarelli D, Pucci A, Gagliardi AM, Mitello L, Montagna A, Latina R (2021). Prevalence and risk factors of delirium in the intensive care unit: an observational study. Nurs Critic Care.

[16] Lee H, Lim CW, Hong HP, Ju JW, Jeon YT, Hwang JW (2015). Efficacy of the APACHE II score at ICU discharge in predicting post-ICU mortality and ICU readmission in critically ill surgical patients. Anaesth Intensive Care.

[17] van den Boogaard M, Peters SA, van der Hoeven JG, Dagnelie PC, Leffers P, Pickkers P, Schoonhoven L (2010). The impact of delirium on the prediction of in-hospital mortality in intensive care patients. Critic Care.

[18] Chen H, Mo L, Hongjuan Hu, Yulan Ou, Luo J (2021). Risk factors of postoperative delirium after cardiac surgery: a meta-analysis. J Cardiothorac Surg.

[19] Aldecoa C, Bettelli G, Bilotta F, Sanders RD, Audisio R, Borozdina A (2017). European society of anaesthesiology evidence-based and consensus based guideline on postoperative delirium. Eur J Anaesthesiol.

[20] Hellmuth R Muller Moran, Duncan Maguire, Doug Maguire, Stephen Kowalski, Eric Jacobsohn, Scott Mackenzie, et al: Association of earlier extubation and postoperative delirium after coronary artery bypass grafting. J Thorac Cardiovasc Surg 2019;19: 30721–4. 10.1016/j.jtcvs.2019.03.047 31076177

[21] Jawa RS, Anillo S, Huntoon K, Baumann H, Kulaylat M (2011). Analytic review: Interleukin-6 in surgery, trauma, and critical care: part I: basic science. J Intensive Care Med.

[22] Rudolph JL, Ramlawi B, Kuchel GA, McElhaney JE, Xie D, Sellke FW (2008). Chemokines are associated with delirium after cardiac surgery. J Gerontol A Biol Sci Med Sci.

[23] Zhang S, Ji M-H, Ding S, Ying Wu, Feng X-W, Tao X-J (2022). Inclusion of interleukin-6 improved performance of postoperative delirium prediction for patients undergoing coronary artery bypass graft (POD-CABG): a derivation and validation study. J Cardiol.

[24] Lv X-C, Lin Y, Qing-Song Wu, Wang L, Hou Y-T, Dong Yi (2021). Plasma interleukin-6 is a potential predictive biomarker for postoperative delirium among acute type a aortic dissection patients treated with open surgical repair. J Cardiothorac Surg.

[25] Chen X, Bai M, Zhao L, Li Y, Yan Yu, Zhang W (2020). Characteristics and outcomes of stanford type A aortic dissection patients with severe post-operation hyperbilirubinemia: a retrospective cohort study. J Cardiothorac Surg.

[26] Dubois MJ, Bergeron N, Dumont M, Dial S, Skrobik Y (2001). Delirium in an intensive care unit: a study of risk factors. Intensive Care Med.

[27] Mancuso C (2017). Bilirubin and brain: a pharmacological approach. Neuropharmacology.

[28] Jon F (2013). Watchko, Claudio Tiribelli: Bilirubin-induced neurologic damage-mechanisms and management approaches. N Engl J Med.

[29] Ghersi-Egea JF, Gazzin S, Strazielle N (2009). Blood-brain interfaces and bilirubin-induced neurological diseases. Curr Pharm Des.

[30] Jäckel M, Aicher N, Rilinger J, Bemtgen X, Widmeier E, Wengenmayer T (2021). Incidence and predictors of delirium on the intensive care unit in patients with acute kidney injury, insight from a retrospective registry. Sci Rep.

[31] D'Hooge R, Van de Vijver G, Van Bogaert P-P, Marescau B, Vanholder R (2003). Peter P De Deyn: involvement of voltage- and ligand-gated Ca2^+^ channels in the neuroexcitatory and synergistic efects of putative uremic neurotoxins. Kidney Int.

[32] Ouimet S, Kavanagh BP, Gottfried SB, Skrobik Y (2007). Incidence, risk factors and consequences of ICU delirium. Intensive Care Med.

[33] Magnusson KR, Scanga C, Wagner AE, Dunlop C (2000). Changes in anesthetic sensitivity and glutamate receptors in the aging canine brain. J Gerontol A Biol Sci Med Sci.

[34] Praticò C, Quattrone D, Lucanto T, Amato A, Penna O, Roscitano C (2005). Drugs of anesthesia acting on central cholinergic system may cause post-operative cognitive dysfunction and delirium. Med Hypotheses.

[35] Pulak LM, Jensen L (2016). Sleep in the intensive care unit: a review. J Intensive Care Med.

